# Long-Term Speech Outcomes After Primary Palatoplasty: A Comparison of Minimal-Incision Intravelar and Modified Veau–Wardill–Kilner Techniques

**DOI:** 10.3390/jcm15197727

**Published:** 2026-10-07

**Authors:** Kostadin Gigov, Ivan Ginev, Petra Kavradzhieva, Mariya Miteva

**Affiliations:** 1 Section of Plastic Reconstructive and Aesthetic Surgery and Thermal Trauma, Department of Propedeutics of Surgical Diseases, Faculty of Medicine, Medical University of Plovdiv “St. George” University Hospital Plovdiv, Peshtersko Shausse Blvd 66, 4002 Plovdiv, Bulgaria; kostadin.gigov@phd.mu-plovdiv.bg (K.G.); ivan.ginev@mu-plovdiv.bg (I.G.); kavradjieva@gmail.com (P.K.); 2 Department of Endocrinology, Faculty of Medicine, Medical University of Plovdiv “St. George” University Hospital Plovdiv, Vassil Aprilov Blvd 15A, 4000 Plovdiv, Bulgaria

**Keywords:** cleft palate, palatoplasty, minimal-incision technique, Veau–Wardill–Kilner technique, speech outcomes, consonant articulation, resonance

## Abstract

**Background/Objectives:** Speech outcome is a major functional endpoint following primary palatoplasty. This study compared long-term speech outcomes after closed intravelar minimal-incision palatoplasty and the modified Veau–Wardill–Kilner procedure. **Methods:** This retrospective observational study evaluated speech using a standardized perceptual protocol adapted to the Bulgarian phonetic system across eight domains. Outcomes were compared between surgical groups, with additional analyses stratified by age and cleft type. **Results:** The minimal-incision group showed higher unadjusted proportions of normal speech intelligibility (29.4% vs. 17.6%, *p* = 0.020), normal resonance (73.2% vs. 54.9%, *p* = 0.001), and normal consonant articulation (37.8% vs. 19.0%, *p* < 0.001), whereas compensatory articulation was less frequent (49.6% vs. 65.5%, *p* = 0.008). Hypernasality, voice quality, and vowel articulation were comparable between groups. After adjustment for cleft type and age group, no significant association was observed between surgical technique and normal speech intelligibility (aOR 1.33, 95% CI 0.73–2.43; *p* = 0.357), whereas the minimal-incision technique was associated with higher odds of normal consonant articulation (aOR 1.79, 95% CI 1.01–3.18; *p* = 0.047) and normal resonance (aOR 1.73, 95% CI 1.02–2.94; *p* = 0.043). **Conclusions:** Both techniques were associated with broadly comparable long-term functional speech outcomes across most evaluated domains. After adjustment for cleft type and age group, differences remained in normal consonant articulation and normal resonance, whereas no significant adjusted association was observed for normal speech intelligibility. These findings should be interpreted cautiously given the historical treatment patterns, non-random distribution of cleft type between groups, and potential residual confounding.

## 1. Introduction

Cleft lip and/or palate is among the most common congenital craniofacial anomalies and may have substantial functional consequences for feeding, hearing, speech development, craniofacial growth, and psychosocial well-being. One of the principal goals of primary palatoplasty is to establish an anatomically and functionally competent palate that enables the development of intelligible speech while minimizing adverse effects on maxillary growth. Nevertheless, speech disorders remain an important concern after palatal repair and may include abnormal resonance, audible nasal emission, reduced speech intelligibility, voice disorders, and articulation errors, including compensatory articulation patterns [1,2].

Postoperative speech outcomes are influenced by multiple factors, including cleft characteristics, timing of palatal closure, velopharyngeal function, surgical technique, and subsequent speech therapy. Patient-related factors, particularly cleft phenotype, have also been associated with postoperative velopharyngeal function and the requirement for secondary speech surgery following primary palatoplasty [3]. However, the relative contribution of the surgical procedure itself remains difficult to establish because comparative studies differ considerably in surgical protocols, patient populations, timing of repair, and methods used for speech assessment. A systematic review of 35 studies including 10,795 patients similarly demonstrated substantial variability in postoperative velopharyngeal outcomes and highlighted heterogeneity in cleft characteristics, timing of repair, surgical technique, and outcome reporting across the available literature [4]. Systematic evidence comparing different methods of palatal closure has also demonstrated variation in speech outcomes between surgical approaches, while emphasizing the need for stronger comparative evidence [5]. The Scandcleft randomized trials demonstrated that surgical protocols may influence specific speech outcomes, although differences were not consistent across all comparisons [6,7]. More recent evidence similarly indicates that the available data do not support a single surgical protocol as universally superior across all speech outcomes [8].

Standardized assessment is therefore essential when comparing functional outcomes after cleft palate repair. Validated perceptual assessment systems have been developed to improve the consistency and reproducibility of cleft-speech evaluation [9,10]. Henningsson et al. proposed universal parameters for reporting cleft-related speech outcomes, including hypernasality, hyponasality, audible nasal air emission, consonant production errors, voice disorders, and overall speech understandability [2]. Such a multidimensional approach is particularly relevant when comparing surgical techniques, as overall speech intelligibility alone may not identify specific differences in resonance or articulation.

The modified Veau–Wardill–Kilner technique has been widely used for primary palatal repair. Anatomical reconstruction of the palatal musculature, particularly through intravelar veloplasty, has become an important component of palatal repair because of its potential relevance to velopharyngeal function and subsequent speech outcomes [11]. In our center, a closed intravelar palatoplasty performed through a minimal-incision approach has also been employed as a tissue-preserving alternative to the modified Veau–Wardill–Kilner technique. In a previous analysis of the same clinical cohort, this minimal-incision approach was associated with a lower incidence of postoperative oro-nasal fistulas and a reduced need for secondary corrective procedures [12]. Whether these surgical advantages are accompanied by comparable or improved long-term functional speech outcomes remains to be established.

Therefore, the present study aimed to compare postoperative speech outcomes following primary palatoplasty using a closed intravelar minimal-incision technique and the modified Veau–Wardill–Kilner technique. Speech intelligibility, resonance, audible nasal emission, facial grimacing, voice quality, vowel articulation, consonant articulation, and recommendations for speech therapy were evaluated, with additional analyses according to age and cleft type. We hypothesized that postoperative speech outcomes would differ between the two surgical techniques across selected speech domains.

## 2. Materials and Methods

### 2.1. Study Design and Population

This retrospective, single-center comparative study evaluated long-term speech outcomes in patients with cleft lip and palate (CLP) or isolated cleft palate (CP) who underwent primary palatoplasty at the Department of Plastic, Reconstructive and Aesthetic Surgery for Children, University Hospital “St. George”, Plovdiv, Bulgaria. The study population represents a subset of a previously described surgical cohort treated between 2005 and 2022, in which postoperative outcomes following two different techniques of primary palatoplasty were evaluated.

Patients were grouped according to the technique used for primary palatal repair: (1) minimal-incision palatoplasty and (2) modified Veau–Wardill–Kilner palatoplasty. Primary palatoplasty was performed between 9 and 12 months of age according to the institutional treatment protocol.

For the present study, postoperative speech evaluations were available for 329 patients, including 178 patients treated with the minimal-incision technique and 151 treated with the modified Veau–Wardill–Kilner technique. Speech outcomes were evaluated at long-term follow-up in patients aged 4–15 years. Because data were incomplete for some individual speech outcome domains, each outcome was analyzed using available cases for that specific domain. Consequently, the number of evaluable patients varied across speech outcomes. Analyses were performed overall and, where appropriate, according to age group and cleft type (CLP versus isolated CP).

### 2.2. Surgical Techniques

Two techniques of primary palatoplasty were compared: closed intravelar palatoplasty with minimal incisions and modified Veau–Wardill–Kilner pushback palatoplasty. Primary palatoplasty was performed between 9 and 12 months of age in all patients. The modified Veau–Wardill–Kilner technique was used throughout the study period, whereas the minimal-incision technique was introduced in 2011; thus, the two surgical groups were only partially contemporaneous.

Closed intravelar palatoplasty with minimal incisions was performed without lateral relaxing incisions and included mobilization of the palatal tissues, palatal lengthening, and anatomical reconstruction of the *levator veli palatini* muscle sling. The modified Veau–Wardill–Kilner pushback procedure involved anterior and lateral relaxing incisions, V–Y transposition of mucoperiosteal flaps for palatal elongation, and posterior repositioning of the *levator veli palatini* musculature.

A detailed description of both surgical procedures, including the technical modifications employed in our center, has been reported previously [12].

### 2.3. Electronic Medical Registry and Follow-Up

Clinical and follow-up data were obtained from the Electronic Medical Register for Facial Anomalies (EMRFA), a national electronic database used for longitudinal follow-up of patients with congenital facial anomalies [12]. The registry contains standardized demographic, diagnostic, surgical, and functional outcome data entered prospectively by members of the multidisciplinary cleft team.

For the present study, demographic and surgical data together with postoperative speech assessments were extracted from the registry. Speech evaluations are recorded in structured fields as part of routine multidisciplinary follow-up. Data used for the present analysis were anonymized prior to analysis. A detailed description of the EMRFA registry and its use for longitudinal outcome assessment has been reported previously [12].

### 2.4. Speech Evaluation

Postoperative speech outcomes were assessed at the latest available follow-up, with one speech assessment per patient included in the present retrospective analysis. The study was not designed as a longitudinal repeated-measures analysis; therefore, a single assessment per patient was used for the between-group comparison. Two experienced speech therapists specialized in the evaluation and rehabilitation of patients with cleft lip and palate independently assessed each patient using the same standardized perceptual speech assessment protocol. In cases of disagreement between the two assessors, the findings were discussed, and a consensus rating was reached. Standardized perceptual protocols for cleft-speech assessment have previously demonstrated acceptable inter- and intra-rater reliability when used by appropriately trained assessors [9]. Because the analytical dataset contained the final consensus ratings rather than the original individual assessor ratings, formal inter-rater reliability could not be calculated retrospectively. The assessment was based on the Eurocleft speech assessment framework adapted to the Bulgarian phonetic system.

Assessments were performed live in a dedicated speech therapy room and were also recorded, with the recordings retained in the patients’ electronic medical records. The speech therapists were not blinded to the surgical technique.

The Bulgarian adaptation accounted for language-specific phonetic and articulatory characteristics. Speech samples included age-appropriate words and sentences of increasing complexity. In children older than 5 years, a standardized text incorporating all Bulgarian consonants and vowels was additionally used. Articulation was assessed during the production of isolated sounds, syllables, words, and complete sentences, whereas speech intelligibility was evaluated during spontaneous speech. Resonance, audible nasal emission, and other perceptual speech characteristics were assessed according to the adapted protocol.

Speech assessment preceded any subsequent secondary pharyngoplasty and formed part of the clinical evaluation used to determine the need for secondary surgical management.

### 2.5. Speech Outcome Measures

Eight speech-related outcome domains were analyzed: (1) speech intelligibility, (2) resonance, (3) audible nasal emission, (4) facial grimacing, (5) voice quality, (6) vowel articulation, (7) consonant articulation, and (8) recommendations for speech therapy. Each domain was classified according to predefined categories of the speech assessment protocol, as summarized in Table 1.

### 2.6. Handling of Missing Speech Data

Missing speech assessment data were not imputed. For each outcome domain, analyses were performed using available cases only, with patients lacking data for the respective outcome excluded from the denominator. Consequently, the number of evaluable patients varied slightly across speech domains and was reported for each analysis, where applicable.

### 2.7. Statistical Analysis

Statistical analyses were performed using SPSS Statistics (version 26.0; IBM Corp., Armonk, NY, USA). Categorical variables were presented as frequencies and percentages [*n* (%)]. Comparisons of individual speech outcome categories between the two surgical groups were performed using Z-tests for comparison of proportions. This approach was chosen to compare the proportion of patients within each clinically defined speech category between techniques, rather than to assume a common ordinal structure across the heterogeneous multidimensional speech outcomes. Fisher’s exact test was used when cell counts were small. Differences in the distributions of age groups and cleft types between the two surgical groups were assessed using the chi-square test. Comparisons between the two surgical techniques were performed for each speech outcome in the corresponding evaluable sample and separately according to age group (4–7 years, 8–12 years, and >12 years). Additional analyses were stratified by cleft type—cleft lip and palate and isolated cleft palate—within each age group. For these age- and cleft-specific analyses, two-sided Fisher’s exact test was used because of the small numbers of patients in several cells. All statistical tests were two-sided, and a *p*-value < 0.05 was considered statistically significant.

Additional multivariable logistic regression analyses were performed using the available stratified aggregate data to account for potential confounding related to the marked imbalance in cleft phenotype between the surgical groups. Separate models were constructed for the selected speech outcomes, with surgical technique as the exposure of interest and cleft type (cleft lip and palate vs. isolated cleft palate) and age group at speech assessment (4–7, 8–12, and >12 years) included as covariates. The modified Veau–Wardill–Kilner technique, CLP, and the 4–7-year age group served as reference categories. Results are presented as adjusted odds ratios (aORs) with 95% confidence intervals (CIs) and corresponding *p*-values. Analyses of normal speech intelligibility, normal consonant articulation, and normal resonance were considered the principal adjusted analyses; additional analyses of specific speech characteristics were considered exploratory and were interpreted cautiously in view of multiple comparisons.

### 2.8. Ethical Considerations

The study was approved by the Scientific Ethics Committee of the Council for Scientific Research at Medical University–Plovdiv (Protocol No. 123/28.04.2023). All procedures were conducted in accordance with the Declaration of Helsinki, and written informed consent was obtained from all patients’ parents or legal guardians.

## 3. Results

### 3.1. Study Population and Patient Characteristics

A total of 329 patients underwent postoperative speech evaluation, including 178 patients treated with the minimal-incision technique and 151 treated with the modified Veau–Wardill–Kilner technique. The distribution across age groups did not differ significantly between the two surgical groups (*p* = 0.122). In contrast, cleft-type distribution differed significantly between groups (*p* < 0.001), with isolated cleft palate being more frequently represented in the minimal-incision group and cleft lip and palate in the modified Veau–Wardill–Kilner group (Table 2). Because data were incomplete for some individual speech outcome domains, the number of evaluable patients varied across the domain-specific analyses.

### 3.2. Speech Intelligibility

In the unadjusted analysis, normal speech was more frequent in the minimal-incision group, whereas speech classified as different but intelligible was more frequent in the modified Veau–Wardill–Kilner group. The more severely impaired intelligibility categories did not differ significantly between groups (Table 3).

In age-stratified analysis, normal speech was more frequent following the minimal-incision technique among patients aged 4–7 years (*p* = 0.019), whereas no significant differences were observed in the older age groups (Supplementary Table S1). Further stratification by age group and cleft type revealed no significant between-technique differences (Supplementary Table S2).

### 3.3. Resonance

Normal resonance was more frequent in the minimal-incision group, whereas hyponasality was more frequent following the modified Veau–Wardill–Kilner procedure. Hypernasality and cul-de-sac resonance did not differ significantly between groups (Table 3).

Age-stratified analyses showed differences in normal resonance and hyponasality in selected age groups, primarily among younger patients (Supplementary Table S3). After further stratification by cleft type, normal resonance was more frequent following the minimal-incision technique among patients with isolated cleft palate aged 4–7 years (*p* = 0.013), with no other significant between-technique differences (Supplementary Table S4).

### 3.4. Audible Nasal Emission

The overall distribution of audible nasal emission was comparable between the two surgical groups, with no significant differences across severity categories (Table 3).

Exploratory stratified analyses identified differences in selected age- and cleft-specific subgroups, whereas no consistent between-technique pattern was observed across age groups (Supplementary Tables S5 and S6).

### 3.5. Facial Grimacing

Facial grimacing was absent in the majority of patients in both groups. Nostril flaring was more frequent in the minimal-incision group, whereas the other facial-movement categories did not differ significantly between techniques (Table 3).

In exploratory stratified analyses, the difference in nostril flaring was observed among patients older than 12 years and within the corresponding isolated cleft palate subgroup; no other significant between-group differences were identified (Supplementary Tables S7 and S8).

### 3.6. Voice Quality

Normal voice quality predominated in both surgical groups, with no significant between-group differences across the evaluated categories (Table 4). No significant differences were identified after stratification by age group or by both age group and cleft type (Supplementary Tables S9 and S10).

### 3.7. Vowel Articulation

Normal vowel articulation predominated in both surgical groups, with no significant between-group differences across the evaluated categories (Table 4). No significant differences were observed after stratification by age group or by both age group and cleft type (Supplementary Tables S11 and S12).

### 3.8. Consonant Articulation

In the unadjusted analysis, normal consonant articulation was more frequent in the minimal-incision group, whereas compensatory articulation was more frequent in the modified Veau–Wardill–Kilner group. Structurally determined articulation errors, with or without associated VPI, did not differ significantly between groups (Table 4).

Age-stratified analyses showed a higher proportion of normal consonant articulation following the minimal-incision technique in the 4–7-year and 8–12-year groups, while compensatory articulation was more frequent following the modified Veau–Wardill–Kilner procedure among patients aged 4–7 years. No significant differences were observed among patients older than 12 years (Supplementary Table S13). Further stratification by both age group and cleft type revealed no significant between-technique differences (Supplementary Table S14).

### 3.9. Recommendations for Speech Therapy

Age-stratified analysis showed differences in speech therapy recommendations between the surgical groups, primarily among younger patients, with no significant differences observed in those older than 12 years (Supplementary Table S15). Further stratification by cleft type localized these differences to specific CP and CLP subgroups (Supplementary Table S16).

### 3.10. Adjusted Analysis of Postoperative Speech Outcomes

In multivariable analyses adjusted for cleft type and age group, the minimal-incision technique was associated with higher odds of normal consonant articulation (aOR 1.79, 95% CI 1.01–3.18; *p* = 0.047) and normal resonance (aOR 1.73, 95% CI 1.02–2.94; *p* = 0.043), whereas no significant adjusted association was observed for normal speech intelligibility (aOR 1.33, 95% CI 0.73–2.43; *p* = 0.357) (Table 5). In exploratory adjusted analyses, no significant associations with surgical technique were observed for compensatory consonant articulation, hypernasality, audible nasal emission, facial grimacing, voice, vowel articulation, or speech-therapy recommendation; however, the minimal-incision technique was associated with lower odds of hyponasality (aOR 0.25, 95% CI 0.09–0.70; *p* = 0.008) (Table S17).

Following speech assessment, secondary pharyngoplasty was performed in 7/178 patients (3.9%) in the minimal-incision group and 21/151 patients (13.9%) in the modified Veau–Wardill–Kilner group (*p* = 0.001). 

## 4. Discussion

The present study provides a multidimensional assessment of postoperative speech outcomes following primary palatoplasty using a closed intravelar minimal-incision technique and the modified Veau–Wardill–Kilner technique. Overall, the two surgical approaches produced broadly comparable outcomes across several speech domains. However, this comparability was not uniform across all speech characteristics. In the unadjusted analyses, the clearest differences were observed in consonant articulation, with normal articulation being more frequent and compensatory articulation less frequent following the minimal-incision technique. After adjustment for cleft type and age group, the association with normal consonant articulation persisted, whereas the difference in compensatory articulation was attenuated and was no longer statistically significant. Normal resonance also remained associated with the minimal-incision technique after adjustment, while the difference in normal speech intelligibility was no longer statistically significant. These findings indicate that some of the crude between-group differences were influenced by differences in cohort composition and emphasize the need for cautious interpretation of technique-related associations.

Speech outcome after palatoplasty is inherently multidimensional, reflecting the interaction between velopharyngeal function, resonance, nasal airflow, articulation, and speech development [2]. The Scandcleft studies demonstrated that differences between surgical protocols may become apparent in specific components of speech without establishing consistent superiority of one protocol across all speech outcomes [6,7,13]. Similarly, a systematic review comparing different methods of cleft palate closure found considerable heterogeneity in reported speech outcomes and highlighted the difficulty of drawing definitive conclusions regarding the superiority of individual surgical techniques [5].

Speech intelligibility represents an integrated functional outcome influenced by several components of speech. Previous comparative studies of palatal repair protocols incorporating different approaches to intravelar veloplasty have likewise evaluated speech intelligibility, nasal air emission, and velopharyngeal function [14,15]. In the present cohort, normal speech intelligibility was more frequent following the minimal-incision technique, whereas the more severely impaired intelligibility categories did not differ significantly between groups. However, after adjustment for cleft type and age group, surgical technique was no longer significantly associated with normal speech intelligibility (aOR 1.33, 95% CI 0.73–2.43; *p* = 0.357). This attenuation suggests that the unadjusted difference was at least partly related to differences in cohort composition rather than to surgical technique alone.

Among the individual speech domains, consonant articulation showed the clearest differences between the surgical groups. Normal consonant articulation was more frequent following the minimal-incision technique, whereas compensatory articulation was less frequent. After adjustment for cleft type and age group, the association with normal consonant articulation remained statistically significant, whereas the association with compensatory articulation was attenuated and no longer reached statistical significance. No significant differences were observed in structurally determined articulation errors, either with or without associated velopharyngeal insufficiency. Compensatory articulation may develop as a learned adaptation and, once established, may persist and require targeted speech therapy [16]. Previous studies of intravelar veloplasty and the Scandcleft trials have also shown that surgical protocols may influence consonant proficiency and cleft-related consonant errors, although these effects are not consistent across all protocols or outcomes [7,11,13]. Taken together, the findings suggest differences in selected articulatory outcomes but should not be interpreted as evidence of superior velopharyngeal competence following the minimal-incision procedure.

Resonance outcomes showed a mixed pattern between the two surgical groups. Normal resonance was more frequently observed following the minimal-incision technique, whereas hyponasality was more frequent after the modified Veau–Wardill–Kilner procedure. After adjustment for cleft type and age group, the minimal-incision technique remained associated with higher odds of normal resonance (aOR 1.73, 95% CI 1.02–2.94; *p* = 0.043), although the proximity of the confidence interval to the null warrants cautious interpretation. The underlying cause of the higher frequency of hyponasality in the modified Veau–Wardill–Kilner group could not be determined retrospectively, as potential contributing factors such as upper airway obstruction, adenoidal hypertrophy, or postoperative scarring were not systematically assessed. As speech assessment preceded secondary pharyngoplasty, overcorrection related to this procedure cannot account for the observed difference. In contrast, hypernasality did not differ significantly between the techniques, and differences in audible nasal emission were not consistently observed across age groups. Postoperative velopharyngeal outcomes vary considerably across published studies and may be influenced by cleft characteristics, timing of repair, and surgical technique [4]. The need for secondary speech-correcting surgery after primary palatoplasty varies across published cohorts and may differ according to the primary surgical approach [17,18]. Taken together, these findings indicate differences in selected resonance characteristics rather than a consistent advantage of either technique in velopharyngeal function.

The remaining perceptual speech domains showed fewer differences between the surgical groups. Voice quality and vowel articulation were predominantly normal and did not differ significantly between techniques. Facial grimacing was also absent in the majority of patients, although nostril flaring was more frequent in the minimal-incision group in selected analyses. Facial movements during speech may occur as secondary behaviors associated with abnormal nasal airflow and should not be interpreted in isolation as a direct measure of velopharyngeal competence [19]. Accordingly, the isolated difference in nostril flaring, in the absence of a consistent parallel pattern in resonance or nasal emission, cannot be readily attributed to the surgical technique.

Recommendations for speech therapy differed mainly in the younger age groups and should be interpreted in relation to the type and persistence of the speech disorder rather than as an isolated outcome. Speech therapy has a particularly important role in learned compensatory articulation, whereas speech characteristics resulting from structural velopharyngeal insufficiency may require physical management [16,20]. Because individual exposure and response to speech therapy were not quantified, differences in therapy recommendations cannot be attributed to the primary surgical technique. Longitudinal studies similarly demonstrate that speech outcomes may change throughout childhood and adolescence and may be influenced by speech therapy and secondary velopharyngeal surgery [18,21,22].

The age-stratified findings should also be considered in the context of the historical evolution of surgical practice at our center. The modified Veau–Wardill–Kilner procedure was used throughout the study period, whereas the minimal-incision technique was introduced in 2011 and subsequently became increasingly used. Thus, the two surgical cohorts were only partially contemporaneous. Although both techniques were used during the later study period, a potential influence of temporal changes in clinical management cannot be entirely excluded. Age may also influence speech outcomes through speech maturation, persistence or modification of compensatory articulation patterns, and cumulative exposure to speech therapy and subsequent treatment [13,16,22]. Therefore, age-stratified differences cannot be attributed solely to the primary surgical procedure.

The distribution of cleft types represents an additional important consideration. Although the presence of an associated cleft lip is not itself a direct determinant of velopharyngeal function, cleft phenotype and severity have been considered potential patient-related factors in studies of speech outcomes after palatoplasty [3]. Following its introduction at our center, the minimal-incision technique was initially preferentially applied to patients with isolated cleft palate before its indications were subsequently expanded, resulting in a greater representation of isolated cleft palate in the minimal-incision group. To account for this baseline imbalance, the principal speech outcomes were evaluated in multivariable analyses adjusted for cleft phenotype and age group, with stratified analyses retained as complementary exploratory analyses. The clinical relevance of the distinction between cleft lip and palate and isolated cleft palate for the present speech outcomes should, however, not be overstated, as both groups had palatal involvement requiring primary palatoplasty. Most between-technique differences were no longer statistically significant within these smaller subgroups; however, stratification substantially reduced sample sizes and statistical power. The absence of statistical significance should therefore not be interpreted as evidence of equivalence between the techniques. Previous longitudinal studies of children with isolated cleft palate treated with minimal-incision palatoplasty and muscle reconstruction have similarly shown that long-term speech outcomes may vary with cleft characteristics and that persistent signs of velopharyngeal insufficiency may necessitate secondary velopharyngeal surgery [23].

Overall, the age- and cleft-stratified findings should be considered exploratory, particularly because residual confounding by patient selection, treatment history, and temporal changes in clinical management cannot be excluded.

### Strengths and Limitations

Several strengths of the present study should be acknowledged. Speech was evaluated by experienced speech therapists using a standardized perceptual protocol adapted to the Bulgarian phonetic system, with assessment of multiple speech domains. The relatively large clinical cohort, multivariable analyses, and complementary analyses stratified by age and cleft type allowed a detailed characterization of postoperative speech outcomes. Furthermore, the study complements our previous evaluation of surgical outcomes in the same underlying cohort [12], providing a broader perspective on structural and functional outcomes following the two palatoplasty techniques.

The study also has several limitations. Its retrospective observational design and the historical evolution of surgical practice resulted in non-random treatment allocation, with differences in cleft-type distribution and only partial temporal overlap between the surgical groups. The modified Veau–Wardill–Kilner technique was used throughout the study period, whereas the minimal-incision technique was introduced in 2011; thus, the two surgical cohorts were only partially contemporaneous. Although both techniques were used during the later study period, a potential influence of temporal changes in clinical management cannot be entirely excluded. Multivariable analyses adjusted for cleft type and age group; nevertheless, residual confounding cannot be excluded. Given the number of speech domains and subgroup comparisons examined, findings from exploratory analyses should be interpreted cautiously because of the possibility of type I error related to multiple comparisons. In addition, long-term speech outcomes may have been influenced by speech therapy, for which individual exposure and response were not quantified. Finally, speech assessment was perceptual and non-blinded, and formal inter-rater reliability could not be calculated retrospectively because the analytical dataset contained consensus ratings rather than the original individual assessor ratings. Instrumental measures of velopharyngeal function were not available.

## 5. Conclusions

Given the retrospective, non-random historical allocation of the surgical techniques and the baseline difference in cleft-type distribution between groups, the findings should be interpreted with appropriate caution. Both primary palatoplasty techniques achieved broadly comparable long-term speech outcomes across most evaluated domains, although adjusted analyses identified differences in normal consonant articulation and normal resonance. The present findings support consideration of a tissue-sparing, muscle-oriented palatoplasty in appropriately selected patients, without evidence of a consistent disadvantage in long-term speech outcomes. Further prospective studies with standardized longitudinal speech assessment are warranted to clarify the independent contribution of surgical technique to long-term speech outcomes.

## Figures and Tables

**Table 1:** Speech outcome domains and categories used in the analysis.

Speech Outcome Domain	Categories Used in the Analysis
Speech intelligibility	Normal speech; different but intelligible; unintelligible to unfamiliar listeners; unintelligible speech
Resonance	Normal resonance; hypernasality; hyponasality; cul-de-sac resonance
Audible nasal emission	No audible nasal emission; mild; moderate; severe
Facial grimacing	Absent; nostril flaring; nostril and lip movements; mid- and upper-face movements
Voice quality	Normal voice; weak voice; strained voice; hoarse voice
Vowel articulation	Normal articulation; weakened articulation; persistent nasalization; nasalized vowels with loss of phonetic identity
Consonant articulation	Normal articulation; structurally determined articulation errors; structurally determined articulation errors associated with VPI; compensatory articulation
Recommendations for speech therapy	Speech therapy not required; speech therapy optional; speech therapy recommended after orthodontic and/or surgical treatment; speech therapy required

Abbreviation: VPI, velopharyngeal insufficiency.

**Table 2:** Characteristics of the speech-evaluated cohort according to surgical technique.

Characteristic	Minimal-Incision (*n* = 178)	Modified Veau–Wardill–Kilner (*n* = 151)	*p*
**Age group**			**0.122**
4–7 years	61 (34.3%)	37 (24.5%)	
8–12 years	80 (44.9%)	73 (48.3%)	
>12 years	37 (20.8%)	41 (27.2%)	
**Cleft type**			**<0.001**
CLP	52 (29.2%)	90 (59.6%)	
CP	126 (70.8%)	61 (40.4%)	

Data are presented as *n* (%). CLP, cleft lip and palate; CP, isolated cleft palate. *p*-values were obtained using the chi-square test to compare the distributions of age groups and cleft types between the two surgical groups. A *p*-value < 0.05 was considered statistically significant. The speech-evaluated cohort comprised all patients who underwent postoperative speech assessment; sample sizes for individual speech outcome analyses varied according to the availability of domain-specific assessment data.

**Table 3:** Perceptual speech outcomes according to surgical technique.

Speech Domain/Category	Minimal-Incision, *n* (%)	Modified Veau–Wardill–Kilner, *n* (%)	*p*
**Speech intelligibility**	***n* = 136**	***n* = 142**	
Normal speech	40 (29.4%)	25 (17.6%)	**0.020**
Different but intelligible	78 (57.4%)	103 (72.5%)	**0.008**
Unintelligible to unfamiliar listeners	5 (3.7%)	4 (2.8%)	0.686
Unintelligible speech	13 (9.6%)	10 (7.0%)	0.446
**Resonance**	***n* = 138**	***n* = 142**	
Normal resonance	101 (73.2%)	78 (54.9%)	**0.001**
Hypernasality	26 (18.8%)	29 (20.4%)	0.739
Hyponasality	4 (2.9%)	30 (21.1%)	**<0.001**
Cul-de-sac resonance	7 (5.1%)	5 (3.5%)	0.522
**Audible nasal emission**	***n* = 138**	***n* = 142**	
None	87 (63.0%)	77 (54.2%)	0.134
Mild	37 (26.8%)	43 (30.3%)	0.520
Moderate	9 (6.5%)	17 (12.0%)	0.116
Severe	5 (3.6%)	5 (3.5%)	0.963
**Facial grimacing**	***n* = 138**	***n* = 140**	
Absent	118 (85.5%)	123 (87.9%)	0.600
Nostril flaring	16 (11.6%)	6 (4.3%)	**0.027**
Nostril and lip movements	1 (0.7%)	6 (4.3%)	0.120
Mid- and upper-face movements	3 (2.2%)	5 (3.6%)	0.723

Data are presented as *n* (%). Comparisons between the two surgical groups were performed using the Z-test for comparison of proportions. A *p*-value < 0.05 was considered statistically significant.

**Table 4:** Voice and articulatory outcomes according to surgical technique.

Speech Domain/Category	Minimal-Incision, *n* (%)	Modified Veau–Wardill–Kilner, *n* (%)	*p*
**Voice quality**	***n* = 138**	***n* = 142**	
Normal	109 (79.0%)	106 (74.6%)	0.390
Weak voice	24 (17.4%)	29 (20.4%)	0.517
Strained voice	3 (2.2%)	2 (1.4%)	0.629
Hoarse voice	2 (1.4%)	5 (3.5%)	0.267
**Vowel articulation**	***n* = 138**	***n* = 142**	
Normal articulation	121 (87.7%)	116 (81.7%)	0.164
Weakened articulation	15 (10.9%)	17 (12.0%)	0.772
Persistent nasalization	2 (1.4%)	7 (4.9%)	0.173 ^1^
Nasalized vowels with loss of phonetic identity	0 (0.0%)	2 (1.4%)	0.498 ^1^
**Consonant articulation**	***n* = 135**	***n* = 142**	
Normal articulation	51 (37.8%)	27 (19.0%)	**<0.001**
Structurally determined articulation errors	8 (5.9%)	6 (4.2%)	0.518
Structurally determined articulation errors associated with VPI	9 (6.7%)	16 (11.3%)	0.182
Compensatory articulation	67 (49.6%)	93 (65.5%)	**0.008**

Data are presented as *n* (%). Comparisons between the two surgical groups were performed using the Z-test for comparison of proportions or Fisher’s exact test, as appropriate. ^1^ Fisher’s exact test. VPI, velopharyngeal insufficiency. A *p*-value < 0.05 was considered statistically significant.

**Table 5:** Multivariable analysis of principal postoperative speech outcomes according to surgical technique.

Principal Speech Outcome	*n*	Adjusted OR	95% CI	*p*
Normal speech intelligibility	278	1.33	0.73–2.43	0.357
Normal consonant articulation	277	1.79	1.01–3.18	**0.047**
Normal resonance	280	1.73	1.02–2.94	**0.043**

Data are presented as adjusted odds ratios (aORs) with 95% confidence intervals (CIs) derived from multivariable logistic regression models adjusted for cleft type and age group (4–7, 8–12, and >12 years). The modified Veau–Wardill–Kilner technique was used as the reference category. An aOR > 1 indicates higher odds of the specified outcome in the minimal-incision group. Statistical significance was set at *p* < 0.05. Given the multiple outcomes evaluated, these estimates should be interpreted cautiously.

## Data Availability

The data presented in this study are available on request from the corresponding author. The data are not publicly available due to privacy and ethical restrictions.

## Supplementary Materials

The following supporting information can be downloaded at: https://www.mdpi.com/article/10.3390/jcm15197727/s1. Table S1: Speech intelligibility according to age group and surgical technique; Table S2: Speech intelligibility according to age group, cleft type, and surgical technique; Table S3: Resonance according to age group and surgical technique; Table S4: Resonance according to age group, cleft type, and surgical technique; Table S5: Audible nasal emission according to age group and surgical technique; Table S6: Audible nasal emission according to age group, cleft type, and surgical technique; Table S7: Facial grimacing according to age group and surgical technique; Table S8: Facial grimacing according to age group, cleft type, and surgical technique; Table S9: Voice quality according to age group and surgical technique; Table S10: Voice characteristics according to age group, cleft type, and surgical technique; Table S11: Vowel articulation according to age group and surgical technique; Table S12: Vowel articulation according to age group, cleft type, and surgical technique; Table S13: Consonant articulation according to age group and surgical technique; Table S14: Consonant articulation according to age group, cleft type, and surgical technique; Table S15: Recommendations for speech therapy according to age group and surgical technique; Table S16: Recommendations for speech therapy according to age group, cleft type, and surgical technique; Table S17: Exploratory multivariable analyses of postoperative speech outcomes according to surgical technique.
