# Supplementary material for: Long-Term Speech Outcomes After Primary Palatoplasty: A Comparison of Minimal-Incision Intravelar and Modified Veau–Wardill–Kilner Techniques
Source: J Clin Med. 2026 Oct 7;15(19):7727. doi: 10.3390/jcm15197727 (PMC13665868; doi:10.3390/jcm15197727)
Supplement: Supplementary file 1 [file jcm-15-07727-s001.zip › jcm-4568989-supplementary.pdf]

Supplementary Materials

Long-Term Speech Outcomes after Primary Palatoplasty: A Comparison of Minimal-Incision Intravelar and Modified Veau–Wardill–Kilner Techniques

Supplementary Table S1. Speech intelligibility according to age group and surgical technique

Speech intelligibility	4–7 years			8–12 years			>12 years		
	Minimal-incision (n = 43)	Modified V-W-K (n = 36)	*p*	Minimal-incision (n = 67)	Modified V-W-K (n = 66)	*p*	Minimal-incision (n = 26)	Modified V-W-K (n = 40)	*p*
**Normal speech**	16 (37.2)	5 (13.9)	**0.019**	19 (28.4)	14 (21.2)	0.340	4 (15.4)	7 (17.5)	0.822
**Different but intelligible**	24 (55.8)	27 (75.0)	0.076	41 (61.2)	48 (72.7)	0.158	15 (57.7)	26 (65.0)	0.550
**Unintelligible to unfamiliar listeners**	2 (4.7)	1 (2.8)	0.664	1 (1.5)	1 (1.5)	0.991	1 (3.8)	3 (7.5)	0.543
**Unintelligible speech**	1 (2.3)	3 (8.3)	0.225	6 (9.0)	3 (4.5)	0.311	6 (23.1)	4 (10.0)	0.148

Data are presented as n (%). V-W-K, Veau–Wardill–Kilner. Comparisons between surgical techniques within each age group were performed using the Z-test for comparison of proportions. A p-value < 0.05 was considered statistically significant.

Supplementary Table S2. Speech intelligibility according to age group, cleft type, and surgical technique

Age group / Category	CLP /Minimal-incision	CLP /Modified V-W-K	*p*	CP/ Minimal-incision	CP /Modified V-W-K	*p*
**4–7 years**						
Normal speech	2 (20.0%)	3 (13.6%)	0.637	14 (42.4%)	2 (14.3%)	0.094
Different but intelligible	6 (60.0%)	17 (77.3%)	0.407	18 (54.5%)	10 (71.4%)	0.343
Unintelligible to unfamiliar listeners	2 (20.0%)	1 (4.5%)	0.224	0 (0.0%)	0 (0.0%)	—
Unintelligible speech	0 (0.0%)	1 (4.5%)	1.000	1 (3.0%)	2 (14.3%)	0.208
**8–12 years**						
Normal speech	1 (5.0%)	6 (15.0%)	0.407	18 (38.3%)	8 (30.8%)	0.614
Different but intelligible	19 (95.0%)	32 (80.0%)	0.249	22 (46.8%)	16 (61.5%)	0.328
Unintelligible to unfamiliar listeners	0 (0.0%)	1 (2.5%)	1.000	1 (2.1%)	0 (0.0%)	1.000
Unintelligible speech	0 (0.0%)	1 (2.5%)	1.000	6 (12.8%)	2 (7.7%)	0.703
**>12 years**						

Age group / Category	CLP /Minimal-incision	CLP /Modified V-W-K	*p*	CP/ Minimal-incision	CP /Modified V-W-K	*p*
Normal speech	0 (0.0%)	5 (23.8%)	0.555	4 (20.0%)	2 (10.5%)	0.661
Different but intelligible	4 (66.7%)	11 (52.4%)	0.662	11 (55.0%)	15 (78.9%)	0.176
Unintelligible to unfamiliar listeners	1 (16.7%)	3 (14.3%)	1.000	0 (0.0%)	0 (0.0%)	—
Unintelligible speech	1 (16.7%)	2 (9.5%)	0.545	5 (25.0%)	2 (10.5%)	0.407

Data are presented as n (%). CLP, cleft lip and palate; CP, isolated cleft palate; V-W-K, Veau–Wardill–Kilner. Comparisons between surgical techniques within each age and cleft-type subgroup were performed using two-sided Fisher's exact test. A p-value < 0.05 was considered statistically significant. A dash indicates that statistical comparison was not applicable because no events were observed in either surgical group.

Supplementary Table S3. Resonance according to age group and surgical technique

Resonance	4–7 years			8–12 years			>12 years		
	**Minimal-incision (n = 43)**	**Modified V-W-K (n = 36)**	***p***	**Minimal-incision (n = 69)**	**Modified V-W-K (n = 66)**	***p***	**Minimal-incision (n = 26)**	**Modified V-W-K (n = 40)**	***p***
Normal resonance	36 (83.7%)	21 (58.3%)	**0.022**	49 (71.0%)	36 (54.5%)	0.052	14 (53.8%)	22 (55.0%)	1.000
Hypernasality	5 (11.6%)	5 (13.9%)	1.000	12 (17.4%)	19 (28.8%)	0.152	8 (30.8%)	6 (15.0%)	0.139
Hyponasality	1 (2.3%)	9 (25.0%)	**0.004**	4 (5.8%)	11 (16.7%)	0.056	0 (0.0%)	9 (22.5%)	**0.009**
Cul-de-sac resonance	1 (2.3%)	1 (2.8%)	1.000	4 (5.8%)	0 (0.0%)	0.120	4 (15.4%)	3 (7.5%)	0.420

Data are presented as n (%). V-W-K, Veau–Wardill–Kilner. Comparisons between surgical techniques within each age group were performed using two-sided Fisher's exact test. A p-value < 0.05 was considered statistically significant.

Supplementary Table S4. Resonance according to age group, cleft type and surgical technique.

Age group / Resonance	CLP /Minimal-incision	CLP /Modified V-W-K	*p*	CP /Minimal-incision	CP/ Modified V-W-K	*p*
**4–7 years**	**n = 10**	**n = 22**		**n = 33**	**n = 14**	
Normal resonance	6 (60.0%)	13 (59.1%)	1.000	**30 (90.9%)**	**8 (57.1%)**	**0.013**
Hypernasality	2 (20.0%)	1 (4.5%)	0.224	3 (9.1%)	4 (28.6%)	0.173
Hyponasality	1 (10.0%)	8 (36.4%)	0.210	0 (0.0%)	1 (7.1%)	0.298
Cul-de-sac resonance	1 (10.0%)	0 (0.0%)	0.313	0 (0.0%)	1 (7.1%)	0.298
**8–12 years**	**n = 21**	**n = 40**		**n = 48**	**n = 26**	
Normal resonance	15 (71.4%)	19 (47.5%)	0.105	34 (70.8%)	17 (65.4%)	0.793
Hypernasality	2 (9.5%)	11 (27.5%)	0.187	10 (20.8%)	8 (30.8%)	0.400
Hyponasality	3 (14.3%)	10 (25.0%)	0.512	1 (2.1%)	1 (3.8%)	1.000
Cul-de-sac resonance	1 (4.8%)	0 (0.0%)	0.344	3 (6.3%)	0 (0.0%)	0.548
**>12 years**	**n = 6**	**n = 21**		**n = 20**	**n = 19**	

Age group / Resonance	CLP /Minimal-incision	CLP /Modified V-W-K	*p*	CP /Minimal-incision	CP/ Modified V-W-K	*p*
Normal resonance	2 (33.3%)	11 (52.4%)	0.648	13 (65.0%)	11 (57.9%)	0.748
Hypernasality	2 (33.3%)	1 (4.8%)	0.115	6 (30.0%)	5 (26.3%)	1.000
Hyponasality	0 (0.0%)	8 (38.1%)	0.136	0 (0.0%)	1 (5.3%)	0.487
Cul-de-sac resonance	2 (33.3%)	1 (4.8%)	0.115	1 (5.0%)	2 (10.5%)	0.605

Data are presented as n (%). Comparisons between surgical techniques within the same age and cleft-type subgroup were performed using two-sided Fisher's exact test. CLP, cleft lip and palate; CP, isolated cleft palate; V-W-K, Veau–Wardill–Kilner. A p-value < 0.05 was considered statistically significant.

Supplementary Table S5. Audible nasal emission according to age group and surgical technique

Audible nasal emission	4–7 years			8–12 years			>12 years		
	Minimal-incision	Modified V-W-K	*p*	Minimal-incision	Modified V-W-K	*p*	Minimal-incision	Modified V-W-K	*p*
**No audible nasal emission**	34 (79.1%)	20 (55.6%)	**0.031**	41 (59.4%)	35 (53.0%)	0.491	12 (46.2%)	22 (55.0%)	0.605
**Mild**	6 (14.0%)	10 (27.8%)	0.164	21 (30.4%)	21 (31.8%)	1.000	10 (38.5%)	12 (30.0%)	0.595
**Moderate**	3 (7.0%)	4 (11.1%)	0.696	4 (5.8%)	8 (12.1%)	0.236	2 (7.7%)	5 (12.5%)	0.695
**Severe**	0 (0.0%)	2 (5.6%)	0.204	3 (4.3%)	2 (3.0%)	1.000	2 (7.7%)	1 (2.5%)	0.557

Data are presented as n (%). Comparisons between surgical groups within each age category were performed using Fisher's exact test.

Supplementary Table S6. Audible nasal emission according to age group, cleft type, and surgical technique

Age group / Audible nasal emission	CLP/Minimal-incision	CLP/ Modified V-W-K	*p*	CP/ Minimal-incision	CP/Modified V-W-K	*p*
**4–7 years**	**n = 10**	**n = 22**		**n = 33**	**n = 14**	
Absent	6 (60.0%)	13 (59.1%)	1.000	**28 (84.8%)**	**7 (50.0%)**	**0.025**
Mild, affecting some consonants	2 (20.0%)	7 (31.8%)	0.681	4 (12.1%)	3 (21.4%)	0.410
Moderate, affecting most consonants	2 (20.0%)	2 (9.1%)	0.572	1 (3.0%)	2 (14.3%)	0.208
Severe, active nasal fricative	0 (0.0%)	0 (0.0%)	1.000	0 (0.0%)	2 (14.3%)	0.084

Age group / Audible nasal emission	CLP/Minimal-incision	CLP/ Modified V-W-K	*p*	CP/ Minimal-incision	CP/Modified V-W-K	*p*
**8–12 years**	**n = 21**	**n = 40**		**n = 48**	**n = 26**	
Absent	11 (52.4%)	19 (47.5%)	0.791	30 (62.5%)	16 (61.5%)	1.000
Mild, affecting some consonants	10 (47.6%)	16 (40.0%)	0.596	11 (22.9%)	5 (19.2%)	0.776
Moderate, affecting most consonants	0 (0.0%)	5 (12.5%)	0.154	4 (8.3%)	3 (11.5%)	0.691
Severe, active nasal fricative	0 (0.0%)	0 (0.0%)	1.000	3 (6.3%)	2 (7.7%)	1.000
**>12 years**	**n = 6**	**n = 21**		**n = 20**	**n = 19**	
Absent	1 (16.7%)	12 (57.1%)	0.165	11 (55.0%)	10 (52.6%)	1.000
Mild, affecting some consonants	3 (50.0%)	8 (38.1%)	0.662	7 (35.0%)	4 (21.1%)	0.480
Moderate, affecting most consonants	2 (33.3%)	1 (4.8%)	0.115	**0 (0.0%)**	**4 (21.1%)**	**0.047**
Severe, active nasal fricative	0 (0.0%)	0 (0.0%)	1.000	2 (10.0%)	1 (5.3%)	1.000

Data are presented as n (%). Comparisons were performed between surgical techniques within the same age group and cleft type using two-sided Fisher's exact test. CLP, cleft lip and palate; CP, isolated cleft palate; V-W-K, Veau–Wardill–Kilner. A p-value < 0.05 was considered statistically significant

Supplementary Table S7. Facial grimacing according to age group and surgical technique

Facial grimacing	4–7 years			8–12 years			>12 years		
	Minimal-incision (n = 43)	Modified V-W-K (n = 36)	*p*	Minimal-incision (n = 69)	Modified V-W-K (n = 65)	*p*	Minimal-incision (n = 26)	Modified V-W-K (n = 39)	*p*
Absent	39 (90.7%)	32 (88.9%)	1.000	58 (84.1%)	54 (83.1%)	1.000	21 (80.8%)	37 (94.9%)	0.092
Nostril flaring	2 (4.7%)	3 (8.3%)	0.655	9 (13.0%)	3 (4.6%)	0.131	5 (19.2%)	0 (0.0%)	**0.009**
Nostril and lip movement	0 (0.0%)	1 (2.8%)	0.456	1 (1.4%)	4 (6.2%)	0.205	0 (0.0%)	1 (2.6%)	1.000
Mid- and upper- face movements	2 (4.7%)	0 (0.0%)	0.498	1 (1.4%)	4 (6.2%)	0.205	0 (0.0%)	1 (2.6%)	1.000

Data are presented as n (%). V-W-K, Veau–Wardill–Kilner. Comparisons between surgical techniques within each age group were performed using two-sided Fisher's exact test. A p-value < 0.05 was considered statistically significant.

Supplementary Table S8. Facial grimacing according to age group, cleft type, and surgical technique

Age group / Facial grimacing	CLP/ Minimal-incision	CLP/ Modified V-W-K	*p*	CP/ Minimal-incision	CP/ Modified V-W-K	*p*
**4–7 years**	**n = 10**	**n = 22**		**n = 33**	**n = 14**	
Absent	9 (90.0%)	19 (86.4%)	1.000	30 (90.9%)	13 (92.9%)	1.000
Nostril flaring	0 (0.0%)	2 (9.1%)	1.000	2 (6.1%)	1 (7.1%)	1.000
Nostril and lip movements	0 (0.0%)	1 (4.5%)	1.000	0 (0.0%)	0 (0.0%)	1.000
Mid- and upper-face movements	1 (10.0%)	0 (0.0%)	0.313	1 (3.0%)	0 (0.0%)	1.000
**8–12 years**	**n = 21**	**n = 40**		**n = 48**	**n = 25**	
Absent	18 (85.7%)	35 (87.5%)	1.000	40 (83.3%)	19 (76.0%)	0.535
Nostril flaring	3 (14.3%)	1 (2.5%)	0.113	6 (12.5%)	2 (8.0%)	0.707
Nostril and lip movements	0 (0.0%)	1 (2.5%)	1.000	1 (2.1%)	3 (12.0%)	0.113
Mid- and upper-face movements	0 (0.0%)	3 (7.5%)	0.545	1 (2.1%)	1 (4.0%)	1.000
**>12 years**	**n = 6**	**n = 21**		**n = 20**	**n = 18**	
Absent	6 (100.0%)	20 (95.2%)	1.000	15 (75.0%)	17 (94.4%)	0.184
Nostril flaring	0 (0.0%)	0 (0.0%)	1.000	**5 (25.0%)**	**0 (0.0%)**	**0.048**
Nostril and lip movements	0 (0.0%)	1 (4.8%)	1.000	0 (0.0%)	0 (0.0%)	1.000
Mid- and upper-face movements	0 (0.0%)	0 (0.0%)	1.000	0 (0.0%)	1 (5.6%)	0.474

Data are presented as n (%). Comparisons were performed between surgical techniques within the same age group and cleft type using two-sided Fisher's exact test. CLP, cleft lip and palate; CP, isolated cleft palate; V-W-K, Veau–Wardill–Kilner. A p-value < 0.05 was considered statistically significant.

Supplementary Table S9. Voice quality according to age group and surgical technique

Voice quality	4–7 years			8–12 years			>12 years		
	**Minimal-incision (n = 43)**	**Modified V-W-K (n = 36)**	*p*	**Minimal-incision (n = 69)**	**Modified V-W-K (n = 66)**	*p*	**Minimal-incision (n = 26)**	**Modified V-W-K (n = 40)**	*p*
Normal voice	36 (83.7%)	27 (75.0%)	0.338	54 (78.3%)	50 (75.8%)	0.733	18 (69.2%)	30 (75.0%)	0.601
Weak voice	7 (16.3%)	7 (19.4%)	0.719	12 (17.4%)	13 (19.7%)	0.735	6 (23.1%)	8 (20.0%)	0.768
Strained voice	0 (0.0%)	0 (0.0%)	—	2 (2.9%)	1 (1.5%)	1.000	1 (3.8%)	1 (2.5%)	1.000
Hoarse voice	0 (0.0%)	2 (5.6%)	0.204	1 (1.4%)	2 (3.0%)	0.613	1 (3.8%)	1 (2.5%)	1.000

Data are presented as n (%). Comparisons between surgical techniques within each age group were performed using two-sided Fisher's exact test. V-W-K, Veau–Wardill–Kilner. A p-value < 0.05 was considered statistically significant. The dash indicates that statistical comparison was not applicable because no cases were observed in either surgical group.

Supplementary Table S10. Voice characteristics according to age group, cleft type, and surgical technique

Age group / Voice characteristics	CLP/ Minimal-incision	CLP /Modified V-W-K	*p*	CP/ Minimal-incision	CP/ Modified V-W-K	*p*
**4–7 years**	**n = 10**	**n = 22**		**n = 33**	**n = 14**	
Normal	7 (70.0%)	18 (81.8%)	0.648	29 (87.9%)	9 (64.3%)	0.102
Weak voice	3 (30.0%)	3 (13.6%)	0.346	4 (12.1%)	4 (28.6%)	0.215
Strained voice	0 (0.0%)	0 (0.0%)	-	0 (0.0%)	0 (0.0%)	-
Hoarse voice	0 (0.0%)	1 (4.5%)	1.000	0 (0.0%)	1 (7.1%)	0.298
**8–12 years**	**n = 21**	**n = 40**		**n = 48**	**n = 26**	
Normal	18 (85.7%)	29 (72.5%)	0.342	36 (75.0%)	21 (80.8%)	0.773
Weak voice	2 (9.5%)	9 (22.5%)	0.302	10 (20.8%)	4 (15.4%)	0.758
Strained voice	1 (4.8%)	0 (0.0%)	0.344	1 (2.1%)	1 (3.8%)	1.000
Hoarse voice	0 (0.0%)	2 (5.0%)	0.541	1 (2.1%)	0 (0.0%)	1.000
**>12 years**	**n = 6**	**n = 21**		**n = 20**	**n = 19**	
Normal	4 (66.7%)	18 (85.7%)	0.303	14 (70.0%)	12 (63.2%)	0.741
Weak voice	2 (33.3%)	2 (9.5%)	0.204	4 (20.0%)	6 (31.6%)	0.480
Strained voice	0 (0.0%)	0 (0.0%)	-	1 (5.0%)	1 (5.3%)	1.000
Hoarse voice	0 (0.0%)	1 (4.8%)	1.000	1 (5.0%)	0 (0.0%)	1.000

Data are presented as n (%). Comparisons were performed between surgical techniques within the same age group and cleft type using two-sided Fisher's exact test. CLP, cleft lip and palate; CP, isolated cleft palate; V-W–K, Veau-Wardill–Kilner. A p-value < 0.05 was considered statistically significant. The dash indicates that statistical comparison was not applicable because no cases were observed in either surgical group.

Supplementary Table S11. Vowel articulation according to age group and surgical technique

Vowel articulation	4–7 years			8–12 years			>12 years		
	Minimal-incision (n = 43)	Modified V-W-K (n = 36)	*p*	Minimal-incision (n = 69)	Modified V-W-K (n = 66)	*p*	Minimal-incision (n = 26)	Modified V-W-K (n = 40)	*p*
Normal articulation	38 (88.4%)	30 (83.3%)	0.519	62 (89.9%)	53 (80.3%)	0.118	21 (80.8%)	33 (82.5%)	0.863
Weakened articulation	4 (9.3%)	4 (11.1%)	0.790	6 (8.7%)	9 (13.6%)	0.364	5 (19.2%)	4 (10.0%)	0.298
Persistent nasalization	1 (2.3%)	1 (2.8%)	1.000[1]	1 (1.4%)	4 (6.1%)	0.202[1]	0 (0.0%)	2 (5.0%)	0.515[1]
Nasalized vowels with loss of phonetic identity	0 (0.0%)	1 (2.8%)	0.456[1]	0 (0.0%)	0 (0.0%)	—	0 (0.0%)	1 (2.5%)	1.000[1]

Data are presented as n (%). Comparisons between surgical techniques within each age group were performed using the Z-test for comparison of proportions or Fisher's exact test, as appropriate. ¹ Fisher's exact test.V-W-K, Veau-Wardill–Kilner. A dash indicates that statistical comparison was not applicable because no cases were observed in either group. A p-value < 0.05 was considered statistically significant.

Supplementary Table S12. Vowel articulation according to age group, cleft type, and surgical technique

Age group / Vowel articulation	CLP /Minimal-incision	CLP /Modified V-W-K	*p*	CP/ Minimal-incision	CP /Modified V-W-K	*p*
**4–7 years**	**n = 10**	**n = 22**		**n = 33**	**n = 14**	
Normal articulation	8 (80.0%)	20 (90.9%)	0.572	30 (90.9%)	10 (71.4%)	0.173
Weakened articulation	1 (10.0%)	2 (9.1%)	1.000	3 (9.1%)	2 (14.3%)	0.627
Persistent nasalization	1 (10.0%)	0 (0.0%)	0.313	0 (0.0%)	1 (7.1%)	0.298
Nasalized vowels with loss of phonetic identity	0 (0.0%)	0 (0.0%)	—	0 (0.0%)	1 (7.1%)	0.298
**8–12 years**	**n = 21**	**n = 40**		**n = 48**	**n = 26**	
Normal articulation	20 (95.2%)	32 (80.0%)	0.143	42 (87.5%)	21 (80.8%)	0.503
Weakened articulation	1 (4.8%)	7 (17.5%)	0.243	5 (10.4%)	2 (7.7%)	1.000
Persistent nasalization	0 (0.0%)	1 (2.5%)	1.000	1 (2.1%)	3 (11.5%)	0.121
Nasalized vowels with loss of phonetic identity	0 (0.0%)	0 (0.0%)	—	0 (0.0%)	0 (0.0%)	—
**>12 years**	**n = 6**	**n = 21**		**n = 20**	**n = 19**	
Normal articulation	3 (50.0%)	17 (81.0%)	0.290	18 (90.0%)	16 (84.2%)	0.661
Weakened articulation	3 (50.0%)	4 (19.0%)	0.290	2 (10.0%)	0 (0.0%)	0.487
Persistent nasalization	0 (0.0%)	0 (0.0%)	—	0 (0.0%)	2 (10.5%)	0.231
Nasalized vowels with loss of phonetic identity	0 (0.0%)	0 (0.0%)	—	0 (0.0%)	1 (5.3%)	0.487

Data are presented as n (%). Comparisons between surgical techniques within each age group and cleft type were performed using two-sided Fisher's exact test. CLP, cleft lip and palate; CP, isolated cleft palate; V-W-K,Veau-Wardill–Kilner. A dash indicates that statistical comparison was not applicable because no cases were observed in either surgical group. A p-value < 0.05 was considered statistically significant.

Supplementary Table S13. Consonant articulation according to age group and surgical technique

Consonant articulation	4–7 years			8–12 years			>12 years		
	Minimal-incision (n = 43)	Modified V-W-K (n = 36)	*p*	Minimal-incision (n = 66)	Modified V-W-K (n = 66)	*p*	Minimal-incision (n = 26)	Modified V-W-K (n = 40)	*p*
Normal articulation	18 (41.9%)	5 (13.9%)	**0.006**	26 (39.4%)	15 (22.7%)	**0.039**	7 (26.9%)	8 (20.0%)	0.512
Structurally determined articulation errors	6 (14.0%)	3 (8.3%)	0.434	1 (1.5%)	1 (1.5%)	1.000¹	1 (3.8%)	2 (5.0%)	1.000¹

Consonant articulation	4–7 years			8–12 years			>12 years		
Structurally determined articulation errors associated with VPI	1 (2.3%)	2 (5.6%)	0.589[1]	4 (6.1%)	10 (15.2%)	0.090	4 (15.4%)	3 (7.5%)	0.420[1]
Compensatory articulation	18 (41.9%)	26 (72.2%)	**0.007**	35 (53.0%)	40 (60.6%)	0.380	14 (53.8%)	27 (67.5%)	0.264

Data are presented as n (%). Comparisons between surgical techniques within each age group were performed using the Z-test for comparison of proportions or Fisher's exact test, as appropriate. [1] Fisher's exact test. VPI, velopharyngeal insufficiency; V-W-K, Veau–Wardill–Kilner. A p-value < 0.05 was considered statistically significant.

Supplementary Table S14. Consonant articulation according to age group, cleft type, and surgical technique

Age group / Consonant articulation	CLP /Minimal-incision	CLP/ Modified V-W-K	*p*	CP /Minimal-incision	CP/ Modified V-W-K	*p*
**4–7 years**	**n = 10**	**n = 22**		**n = 33**	**n = 14**	
Normal articulation	2 (20.0%)	2 (9.1%)	0.572	16 (48.5%)	3 (21.4%)	0.111
Structurally determined articulation errors	2 (20.0%)	3 (13.6%)	0.637	4 (12.1%)	0 (0.0%)	0.302
Structurally determined articulation errors associated with VPI	1 (10.0%)	1 (4.5%)	0.534	0 (0.0%)	1 (7.1%)	0.298
Compensatory articulation	5 (50.0%)	16 (72.7%)	0.252	13 (39.4%)	10 (71.4%)	0.060
**8–12 years**	**n = 20**	**n = 40**		**n = 46**	**n = 26**	
Normal articulation	4 (20.0%)	6 (15.0%)	0.718	22 (47.8%)	9 (34.6%)	0.328
Structurally determined articulation errors	1 (5.0%)	1 (2.5%)	1.000	0 (0.0%)	0 (0.0%)	-
Structurally determined articulation errors associated with VPI	0 (0.0%)	7 (17.5%)	0.084	4 (8.7%)	3 (11.5%)	0.698
Compensatory articulation	15 (75.0%)	26 (65.0%)	0.560	20 (43.5%)	14 (53.8%)	0.465
**>12 years**	**n = 6**	**n = 21**		**n = 20**	**n = 19**	
Normal articulation	0 (0.0%)	4 (19.0%)	0.545	7 (35.0%)	4 (21.1%)	0.480
Structurally determined articulation errors	0 (0.0%)	2 (9.5%)	1.000	1 (5.0%)	0 (0.0%)	1.000
Structurally determined articulation errors associated with VPI	1 (16.7%)	0 (0.0%)	0.222	3 (15.0%)	3 (15.8%)	1.000
Compensatory articulation	5 (83.3%)	15 (71.4%)	1.000	9 (45.0%)	12 (63.2%)	0.341

Data are presented as n (%). Comparisons between surgical techniques within each age group and cleft type were performed using two-sided Fisher's exact test. CLP, cleft lip and palate; CP, isolated cleft palate; VPI, velopharyngeal insufficiency; V-W-K, Veau–Wardill–Kilner. The dash indicates that statistical comparison was not applicable because no cases were observed in either group. A p-value < 0.05 was considered statistically significant.

Supplementary Table S15. Recommendations for speech therapy according to age group and surgical technique

Recommendations for speech therapy	4–7 years			8–12 years			>12 years		
	Minimal-incision (n = 45)	Modified V-W-K (n = 36)	*p*	Minimal-incision (n = 69)	Modified V-W-K (n = 66)	*p*	Minimal-incision (n = 27)	Modified V-W-K (n = 40)	*p*
Speech therapy not required	16 (35.6%)	3 (8.3%)	**0.004**	19 (27.5%)	14 (21.2%)	0.392	4 (14.8%)	6 (15.0%)	0.982
Speech therapy optional	0 (0.0%)	6 (16.7%)	**0.007** [1]	6 (8.7%)	6 (9.1%)	0.944	4 (14.8%)	8 (20.0%)	0.589
Speech therapy recommended after orthodontic and/or surgical treatment	6 (13.3%)	5 (13.9%)	0.944	4 (5.8%)	12 (18.2%)	**0.029**	5 (18.5%)	5 (12.5%)	0.503
Speech therapy required	23 (51.1%)	22 (61.1%)	0.366	40 (58.0%)	34 (51.5%)	0.455	14 (51.9%)	21 (52.5%)	0.962

Data are presented as n (%). Comparisons between surgical techniques within each age group were performed using the Z-test for comparison of proportions or Fisher's exact test, as appropriate. [1] Fisher's exact test. V-W-K, Veau–Wardill–Kilner. A p-value < 0.05 was considered statistically significant.

Supplementary Table S16. Recommendations for speech therapy according to age group, cleft type, and surgical technique

Age group / Recommendations for speech therapy	CLP /Minimal-incision	CLP /Modified V-W-K	*p*	CP/Minimal-incision	CP/Modified V-W-K	*p*
**4–7 years**	**n = 11**	**n = 22**		**n = 34**	**n = 14**	
Speech therapy not required	2 (18.2%)	2 (9.1%)	0.586	14 (41.2%)	1 (7.1%)	**0.037**
Speech therapy optional	0 (0.0%)	4 (18.2%)	0.276	0 (0.0%)	2 (14.3%)	0.081
Speech therapy recommended after orthodontic and/or surgical treatment	2 (18.2%)	4 (18.2%)	1.000	4 (11.8%)	1 (7.1%)	1.000
Speech therapy required	7 (63.6%)	12 (54.5%)	0.719	16 (47.1%)	10 (71.4%)	0.202
**8–12 years**	**n = 21**	**n = 40**		**n = 48**	**n = 26**	
Speech therapy not required	1 (4.8%)	6 (15.0%)	0.405	18 (37.5%)	8 (30.8%)	0.618
Speech therapy optional	5 (23.8%)	5 (12.5%)	0.291	1 (2.1%)	1 (3.8%)	1.000
Speech therapy recommended after orthodontic and/or surgical treatment	0 (0.0%)	9 (22.5%)	**0.021**	4 (8.3%)	3 (11.5%)	0.691
Speech therapy required	15 (71.4%)	20 (50.0%)	0.173	25 (52.1%)	14 (53.8%)	1.000
**>12 years**	**n = 6**	**n = 21**		**n = 21**	**n = 19**	
Speech therapy not required	0 (0.0%)	4 (19.0%)	0.545	4 (19.0%)	2 (10.5%)	0.664
Speech therapy optional	2 (33.3%)	6 (28.6%)	1.000	2 (9.5%)	2 (10.5%)	1.000
Speech therapy recommended after orthodontic and/or surgical treatment	1 (16.7%)	2 (9.5%)	0.545	4 (19.0%)	3 (15.8%)	1.000
Speech therapy required	3 (50.0%)	9 (42.9%)	1.000	11 (52.4%)	12 (63.2%)	0.538

Data are presented as n (%). Comparisons between surgical techniques within each age group and cleft type were performed using two-sided Fisher's exact test. CLP, cleft lip and palate; CP, isolated cleft palate; V-W-K, Veau–Wardill–Kilner. A p-value < 0.05 was considered statistically significant.

Supplementary Table S17. Exploratory multivariable analyses of postoperative speech outcomes according to surgical technique

Exploratory speech outcome	n	Adjusted OR	95% CI	*p*
Compensatory consonant articulation	277	0.65	0.39–1.09	0.100
Hypernasality	280	0.71	0.38–1.34	0.286
Hyponasality	280	0.25	0.09–0.70	**0.008**
Absence of audible nasal emission	280	1.21	0.72–2.01	0.472
Absence of facial grimacing	278	0.93	0.45–1.96	0.857
Normal voice	280	1.23	0.68–2.22	0.500

**Exploratory speech outcome**	**n**	**Adjusted OR**	**95% CI**	***p***
Normal vowel articulation	280	1.54	0.76–3.11	0.228
Speech therapy not required	283	1.44	0.78–2.68	0.243

Data are presented as adjusted odds ratios (aORs) with 95% confidence intervals (CIs). Separate multivariable logistic regression models were fitted for each outcome and adjusted for cleft type and age group (4–7, 8–12, and >12 years). The modified Veau–Wardill–Kilner technique was used as the reference surgical category. An aOR > 1 indicates higher odds of the specified outcome in the minimal-incision group, whereas an aOR < 1 indicates lower odds. These analyses were considered exploratory and should be interpreted cautiously in view of multiple comparisons.
